# Suppression of Hydroxycinnamate Network Formation in Cell Walls of Rice Shoots Grown under Microgravity Conditions in Space

**DOI:** 10.1371/journal.pone.0137992

**Published:** 2015-09-17

**Authors:** Kazuyuki Wakabayashi, Kouichi Soga, Takayuki Hoson, Toshihisa Kotake, Takashi Yamazaki, Akira Higashibata, Noriaki Ishioka, Toru Shimazu, Keiji Fukui, Ikuko Osada, Haruo Kasahara, Motoshi Kamada

**Affiliations:** 1 Department of Biological Sciences, Graduate School of Science, Osaka City University, Sumiyoshi-ku, Osaka, Japan; 2 Division of Life Science, Graduate School of Science and Engineering, Saitama University, Sakura-ku, Saitama, Japan; 3 Japan Aerospace Exploration Agency (JAXA), Sengen, Tsukuba, Japan; 4 Japan Space Forum, Kandasurugadai, Chiyoda-ku, Tokyo, Japan; 5 Japan Manned Space Systems Corp., Kawaguchi, Tsuchiura, Japan; 6 Advanced Engineering Services Co., Ltd., Takezono, Tsukuba, Japan; Iwate University, JAPAN

## Abstract

Network structures created by hydroxycinnamate cross-links within the cell wall architecture of gramineous plants make the cell wall resistant to the gravitational force of the earth. In this study, the effects of microgravity on the formation of cell wall-bound hydroxycinnamates were examined using etiolated rice shoots simultaneously grown under artificial 1 *g* and microgravity conditions in the Cell Biology Experiment Facility on the International Space Station. Measurement of the mechanical properties of cell walls showed that shoot cell walls became stiff during the growth period and that microgravity suppressed this stiffening. Amounts of cell wall polysaccharides, cell wall-bound phenolic acids, and lignin in rice shoots increased as the shoot grew. Microgravity did not influence changes in the amounts of cell wall polysaccharides or phenolic acid monomers such as ferulic acid (FA) and *p*-coumaric acid, but it suppressed increases in diferulic acid (DFA) isomers and lignin. Activities of the enzymes phenylalanine ammonia-lyase (PAL) and cell wall-bound peroxidase (CW-PRX) in shoots also increased as the shoot grew. PAL activity in microgravity-grown shoots was almost comparable to that in artificial 1 *g*-grown shoots, while CW-PRX activity increased less in microgravity-grown shoots than in artificial 1 *g*-grown shoots. Furthermore, the increases in expression levels of some class III peroxidase genes were reduced under microgravity conditions. These results suggest that a microgravity environment modifies the expression levels of certain class III peroxidase genes in rice shoots, that the resultant reduction of CW-PRX activity may be involved in suppressing DFA formation and lignin polymerization, and that this suppression may cause a decrease in cross-linkages within the cell wall architecture. The reduction in intra-network structures may contribute to keeping the cell wall loose under microgravity conditions.

## Introduction

Environmental stimuli such as gravity, light, temperature, and water have a fundamental effect on processes in the plant life cycle. Of these stimuli, gravity is always present, and has a constant direction and magnitude. When ancestral plants moved from the sea to the land around 450 million years ago, the development of a tough body to resist the force of gravity was critical for their existence [1, 2]. Terrestrial plants have evolved in the presence of gravity and have developed a cell wall to support their body and keep it upright against gravitational force at 1 *g*, as do the bones and muscles of an animal. The cell wall gives protoplasts structural (mechanical) rigidity and thus directly determines the size and shape of plant cells. Thus, the cell wall plays an important role in the regulation of growth and morphogenesis in plants [3, 4].

Plant cell walls are composed of a variety of polysaccharides, proteins, and phenolic compounds. Based on the differences in matrix polysaccharide composition, plant cell walls are divided into two groups, type I and type II wall [5]. Gramineous (cereal) plants such as rice, wheat, maize, oat, and barley have the type II wall, while most other monocotyledons and dicotyledons have the type I wall [5]. The type I wall contains a large amount of pectic polymers, such as rhamnogalacturonans and arabinogalactans, and xyloglucans. On the other hand, the type II wall contains only a small amount of pectic polymers and xyloglucans, but a large amount of arabinoxylans and β-1,3:1,4-glucans (β-glucans). In addition to cell wall polysaccharides, the type II wall contains a significant amount of phenolic acid monomers such as ferulic acid (FA) and *p*-coumaric acid, which are ester-linked to arabinoxylans [6, 7]. Of these monomers, some FA residues undergo a coupling reaction to produce diferulic acid (DFA), which forms cross-links between arabinoxylans, the major matrix polysaccharides of the type II wall [8, 9]. The ferulate network (cross-linkage of matrix polysaccharides by DFA) forms a tight network within the architecture of the cell wall [5, 10]. DFA and FA levels have indeed been shown to influence the regulation of cell wall strength in gramineous plants [11–16].

Several biochemical steps are involved in the formation of the ferulate network. In shoots of oat, rice, maize, and wheat, the DFA content changed in parallel with the FA content [11–14], suggesting that the rate of the DFA formation is largely dependent on the amount of FA in cell walls. Hydroxycinnamate monomers, including FA and also *p*-coumaric acid, are synthesized in the cytoplasm via the phenylpropanoid pathway, and phenylalanine ammonia-lyase (PAL) catalyzes the first step of the pathway and plays a rate-limiting role in it [17]. Indeed, the change in PAL activity closely correlated with the rate of increase in the amount of wall-bound FA in some gramineous shoots [13–15]. In addition to the amount of wall-bound FA, the coupling process of FA, catalyzed by cell wall-bound peroxidase (CW-PRX), is functionally important in the mechanism of DFA formation [10, 18]. Treatment of maize suspension cells with hydrogen peroxide activated apoplastic peroxidases and thus stimulated the DFA formation [8]. Furthermore, the promotion of DFA formation was found to be closely associated with the increase in CW-PRX activity in segments of oat shoots [19]. We found that an increase in DFA content was found to be closely correlated with increased CW-PRX activity in rice shoots [20]. Therefore, both PAL and CW-PRX play key roles and are deeply involved in the mechanism in regulation of ferulate network formation in gramineous shoots.

We have previously studied the effects of gravity on cell wall properties in plant organs using centrifugation techniques. Centrifugation produces conditions of hypergravity, i.e., the gravitational forces of more than 1 *g*. Hypergravity treatment has been shown to increase the rigidity (mechanical strength) of cell walls [21, 22]. In terms of the chemical properties of cell walls, continuous hypergravity treatment (300 *g* for 2–4 d) stimulated the formation of ferulate networks within the cell wall architecture of dark-grown wheat shoots [23]. Furthermore, hypergravity treatment increased the activity of CW-PRX in the stem tissues of azuki bean seedlings [24]. These results suggest that a hypergravity environment increases CW-PRX activity in stem tissues of terrestrial plants and that in case of gramineous plants, such an increase in CW-PRX activity may stimulate ferulate network formation, resulting in making the firm cell wall structure against the increased gravitational force. From these findings, we hypothesized that, in contrast to hypergravity, an environment of microgravity may reduce the formation of hydroxycinnamate cross-links in gramineous cell walls by affecting activities of enzymes such as PAL and CW-PRX. We carried out a space experiment called Ferulate to examine above hypothesis. In this experiment, we compared the properties of rice shoots grown in space under artificial 1 *g* and microgravity conditions; experiment was conducted at the Cell Biology Experiment Facility (CBEF) on the International Space Station (ISS).

## Materials and Methods

### Plant materials

Rice (*Oryza sativa* L., cv. Koshihikari) was used for the experiment. Three days before the launch, rice caryopses were sterilized in 5% (v/v) sodium hypochlorite solution for 1 h and then thoroughly washed with sterilized water. Twenty-five sterilized caryopses were aligned along a groove on 11 mL of 1% (w/v) agar (Bacto Agar, BD, NJ, USA) in a black polycarbonate culture dish (W × D × H = 40 × 40 × 30 mm, outer dimensions) that shaded caryopses from light irradiation. We prepared 48 culture dishes. After planting, culture dishes were stored in a Measurement Experiment Unit (MEU) and kept at 2–4°C before and during the flight, until the start of the growth experiment on the ISS. One MEU contained 12 culture dishes. In the preliminary experiment, we checked that the storage at 2–4°C prevented germination of planted rice caryopses for one month and that cold-stored caryopses kept their ability to germinate and grow during the storage period. Pictures of culture dish and experimental devices (MEU and CBEF) used in the experiment are shown in S1 Fig

### Onboard procedures

Four MEUs containing rice caryopses were launched aboard Space Shuttle STS-132 on May 15, 2010. In orbit, the MEUs were stored in the Minus-Eighty Laboratory Freezer for ISS (MELFI) at 2°C. Twelve days after the launch, the MEUs were removed and transferred to the CBEF of the Kibo Module. The CBEF is an incubator with two compartments: a microgravity part and an artificial gravity part, in which a centrifuge produces 1 *g* conditions [25]. The CBEF has been shown to regulate thermal environment accurately and has already been used in space experiments on the ISS [25]. Two MEUs were placed in the microgravity compartment of the CBEF (microgravity conditions) and two were attached to the centrifuge in the artificial gravity compartment, which was rotated to produce acceleration at 1 *g* (artificial 1 *g* conditions) (S1 Fig). Temperature inside the CBEF was increased to 22.5°C under microgravity conditions for culture dishes in both compartments. Rotation was started when the temperature inside the MEUs reached 20°C. The temperature of culture dishes increased at the same rate in the microgravity and artificial gravity compartments, reaching 20°C and 22.6°C at around 100 min and 3 h, respectively, after being transferred to the CBEF (S2 Fig). The caryopses were allowed to germinate and grown in darkness for 99 h (4 d), 127 h (5.3 d), or 136 h (5.6 d) at 22.6±0.1°C. At the end of each culture period, eight of culture dishes for each gravity condition were taken out of the MEU, immediately transferred to the MELFI and stored at -80°C. The samples were brought back to earth aboard Space Shuttle STS-133 on March 9, 2011, and transported to the laboratory at Osaka City University. Seedlings were kept frozen during the flight and transportation to the laboratory and were stored at -80°C until the analysis.

### Measurement of mechanical properties of the cell wall

Shoots consisting of the coleoptile and first leaf were excised from the frozen rice seedlings. Excised shoots were immediately boiled for 10 min in 80% (v/v) ethanol and then stored in fresh 80% ethanol until analysis. Before the mechanical properties of the cell wall were measured, the ethanol-fixed shoots were rehydrated for several hours at room temperature in several changes of water. The lengths of shoots were then measured with a ruler. The mechanical properties of the cell wall were measured according to the stress-relaxation method using a tensile tester (RTM-25, Toyo Baldwin, Tokyo, Japan). The subapical region of the shoot (2–3 mm below the tip) was fixed between two clamps 2 mm apart, and stretched by lowering the bottom clamp at a speed of 20 mm min^-1^ until a load of 10 g was achieved. A stress-relaxation parameter, minimum stress-relaxation time (T_0_), was calculated according to the equation of Yamamoto et al. [26].

### Fractionation of cell wall components

The ethanol-fixed shoots were rehydrated and the cell wall components fractionated following the method in Wakabayashi et al. [14]. Rehydrated shoots were homogenized in water, washed successively with water, acetone and a methanol:chloroform mixture (1:1, v/v), and treated with 2 units mL^-1^ porcine pancreatic α-amylase (type I-A, Sigma, St. Louis, MO, USA) in 50 mM sodium acetate buffer (pH 6.5) at 37°C for 3 h. After the amylase treatment, the cell walls were treated with 0.1 M NaOH for 24 h and then with 1 M NaOH for 24 h, in darkness at room temperature under N_2_, to extract ester-linked phenolic acids [27]. Thereafter, the residual cell wall material was extracted in three times (8 h each) with 17.5% NaOH containing 0.02% NaBH_4_ at room temperature. The fraction extracted with 17.5% NaOH was neutralized with acetic acid. After cell wall-bound phenolics had been extracted from the 0.1 and 1 M NaOH solutions (described below), the remaining solution was combined with the 17.5% NaOH extracts. This mixture was designated the matrix polysaccharide fraction. The alkali-insoluble residue (cellulose fraction) was washed successively with 0.03 M acetic acid and ethanol, dried at 40°C, dissolved in 72% sulfuric acid for 1 h at room temperature, and diluted with a 29-fold volume of water. The sugar content in each fraction was determined by the phenol-sulfuric acid method [28] using glucose as the standard.

### Determination of cell wall-bound phenolic constituents

Ester-linked phenolic acids liberated from the cell wall with 0.1 and 1 M NaOH solutions (see above) were extracted three times with ethyl acetate after acidifying the alkali extracts to pH 3 with HCl [20]. The ethyl acetate extracts derived from the 0.1 and 1 M NaOH solutions were combined and dried. The dried samples were stored in darkness at -20°C. The liberated phenolic acids were analyzed using a high-performance liquid chromatography (HPLC) system (LC-6A, Shimadzu, Kyoto, Japan) equipped with an Inertsil ODS-3 column (4.6 × 250 mm, GL Science Inc, Tokyo, Japan) and a photodiode array detector (SPD-M20A, Shimadzu) following the method described by Parr et al. [29] and Waldron et al. [27]. Samples were eluted with methanol at a linearly increasing concentration of 10–73% in 5 mM trifluoroacetic acid (approximately pH 4). Phenolic acid monomers were identified and quantified using *trans*-ferulic acid and *trans*-*p*-coumaric acid as standards (Wako Pure Chemical Industries, Ltd., Osaka, Japan). DFA isomer peaks were identified and quantified using 5–5’–DFA, synthesized according to the method described by Richtzenhain [30], and published spectra and response factors reported by Waldron et al. [27].

Lignin content was determined according to the method of Kamisaka et al. [11]. The alkali-insoluble residue (cellulose fraction) was prepared as described above. The dried residue was dissolved in 2 mL of 25% (v/v) acetyl bromide in glacial acetic acid and kept at 70°C for 30 min. After cooling to room temperature, 2 mL of 2 M NaOH solution was added. The mixture was centrifuged at 1,000 *g* for 10 min, after which 0.2 mL of 7.5 M hydroxylamine was added to the supernatant. The amount of lignin was determined by measuring the absorbance of the final solution at 280 nm. Authentic lignin (Tokyo Kasei Kogyo Co. Ltd., Tokyo) was used as the standard.

### PAL and CW-RX

#### Activity assays

After excising, shoots were immediately frozen with liquid nitrogen and kept at -80°C until use. The activities of PAL and CW-PRX were assayed following the method in Wakabayashi et al. [14, 20, 24]. The frozen shoots (ca. 40–100 mg) were homogenized in a mortar with ice-cold 100 mM potassium borate buffer (pH 8.8) containing 2 mM mercaptoethanol and centrifuged at 10,000 *g* for 10 min at 4°C. After centrifugation, PAL activity was assayed using the supernatant. The reaction mixture (2 mL) contained 0.5 mL of 4 mM L-phenylalanine, 0.5 mL of extract, and 1 mL of 100 mM potassium borate buffer (pH 8.8). The reaction mixture was incubated at 37°C for 60 min, after which the reaction was terminated by adding 0.1 mL of 5 N HCl to the mixture. The mixture was then subjected to extraction with ethyl acetate three times. The extract was dried and kept in darkness at -20°C. The amount of *t*-cinnamic acid produced by PAL was determined using the HPLC and solvent system described above. Enzyme activity was expressed as the amount of *t*-cinnamic acid produced after 1 h of incubation. The protein content in the extract was determined using an assay kit (Protein Assay Kit, Bio-Rad Lab. Inc., Hercules, CA).

For the CW-PRX activity assay, the frozen shoots were homogenized in a mortar with ice-cold 10 mM sodium phosphate buffer (pH 7.0). Cell wall residues were collected on a polypropylene mesh (32 μm pore size) and thoroughly washed with the same buffer. The washed residues were suspended in 10 mM sodium phosphate buffer (pH 6.0) containing 1.5 M NaCl. The suspension was kept for 24 h at 4°C and then centrifuged at 10,000 *g* for 20 min at 4°C. The supernatant was used for the assay. CW-PRX activity was measured using two substrates, guaiacol and FA. For guaiacol, the reaction mixture (2 mL) contained 100 μL of enzyme preparation, 13 mM guaiacol, and 5 mM hydrogen peroxide in 40 mM MES-KOH buffer (pH 6.0). The reaction was initiated by adding hydrogen peroxide at 25°C. Absorbance at 470 nm was recorded at 15–s intervals, and enzyme activity was quantified as the increase in absorbance from 1 to 2 min after hydrogen peroxide was added. When FA was used as a substrate, the reaction mixture (2 mL) contained 50 μL of enzyme preparation, 0.1 mM FA, and 5 mM hydrogen peroxide in 50 mM sodium phosphate buffer (pH 6.0). Enzyme activity was quantified according to the decrease in absorbance at 310 nm from 0.5 to 1.0 min after the addition of hydrogen peroxide at 25°C. Protein content was determined as described above.

#### Quantifying CW-PRX gene expression levels

As above, frozen shoots (ca. 60–100 mg) were homogenized using a mortar and a pestle. Total RNA was extracted using the RNeasy Plant Mini Kit (Qiagen, Valencia, CA, USA), including a DNA elimination step (RNase-Free DNase Set, Qiagen). cDNA was then synthesized from the RNA sample (ca. 200 ng), and then the Cyanine 3 (Cy3)-labeled RNA was synthesized with Cy3-CTP using the Low Input Quick Amp Labeling Kit (Agilent Technologies, Santa Clara, CA, USA) for microarray analysis. The labeled RNA was purified, fragmented, and then hybridized onto the microarray slides (Agilent Rice Oligo Microarray 4 × 44K, Design ID 015058, Agilent Technologies) according to the manufacturer’s instructions. The microarray slides were scanned on the SureScan Microarray Scanner System (G4900DA, Agilent Technologies). Hybridization signals (fluorescence intensities) on the arrays were analyzed with Feature Extraction Software (Agilent Technologies). Microarray analysis was conducted on four independent replicates prepared from four culture dishes. Class III peroxidases are specific to plants and considered as secreted extracellular proteins, and the rice genome contains 138 class III peroxidase genes [31]. We therefore analyzed the expression levels of class III peroxidase genes, using the values of fluorescence intensities obtained through a microarray analysis as described above.

In addition to the microarray analysis, the expression level of class III peroxidase genes was analyzed by the quantitative real-time RT-PCR according to the method of Wakabayashi et al. [20]. Briefly, single strand cDNA was synthesized from ca. 8 μg of total RNA using High Capacity cDNA Reverse Transcription Kit (Applied Biosystems, Foster City, CA, USA) with a random hexamer primer. Real-time RT-PCR was performed with the Applied Biosystems 7500 Real Time PCR System with SYBR Green PCR Master Mix (Applied Biosystems) according to the manufacturer’s instructions. A set of specific primers was designed based on the conserved region of six sequences of rice class III peroxidase genes [20]. Data were normalized with respect to 18S rRNA, which was measured as an internal standard.

### Statistical analysis

For each measurement, the means and the standard errors of the means (SE) were calculated. The significance of differences between artificial (on-orbit) 1 *g* and microgravity conditions was analyzed by the Student’s paired *t*-test. In addition, the significance of differences of T_0_ values among each gravity condition was analyzed by the Tukey’s test.

## Results and Discussion

### Morphology and growth

We have previously found that light irradiation can increase the amount of cell wall-bound phenolic acids in some gramineous plants [13, 16]. Light irradiation may thus oppose and negate the effects of microgravity on the formation of these phenolic acids. Therefore, to focus on the effects solely of the gravitational environment, we used etiolated (dark-grown) seedlings in the present experiment.

**Fig 1:**
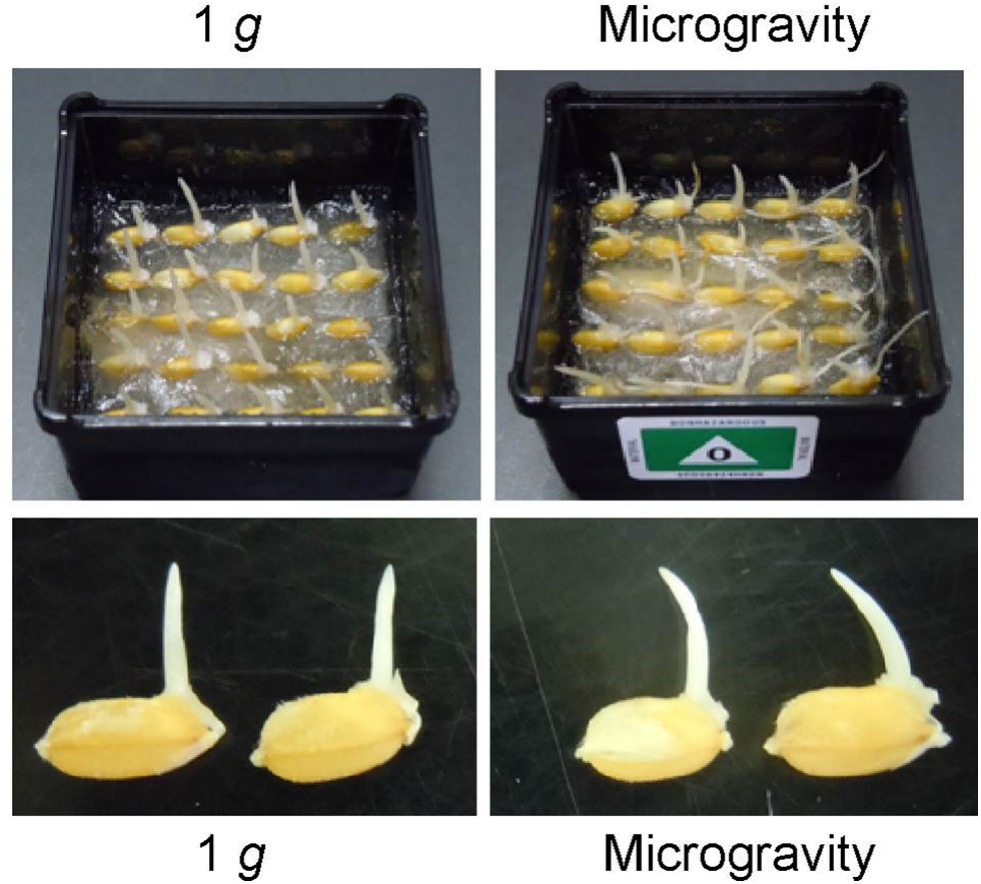
Rice seedlings grown for 4 days under artificial (on-orbit) 1 *g* and microgravity conditions in orbit. Lower panels show magnified images of 4 d seedlings whose roots were excised.

The germination rate of microgravity-grown caryopses, approximately 95%, was almost the same as that of artificial 1 *g*-grown ones. Rice seedlings grown in orbit for 4 d under artificial 1 *g* and microgravity conditions are shown in Fig 1. Under artificial 1 *g* conditions, shoots grew upward and roots elongated downward along the gravity vector. A similar morphology was observed for rice seedlings grown on the ground [32], indicating that the artificial gravity environment in orbit mimics the 1 *g* (control) condition on the ground. In contrast, under microgravity conditions, shoots were not straight but bent toward their caryopses and roots grew in various directions with some emerging into the air (Fig 1). In other words, rice seedlings show automorphogenesis under microgravity conditions in space, as previously reported [32].

Rice shoots grew substantially during the cultivation period; the lengths of shoots grown under artificial 1 *g* and microgravity conditions were 6.7±0.3 and 6.3±0.3 mm at 4 d (n = 25), 12.6±0.3 and 12.7±0.3 mm at 5.3 d (n = 35), and 16.9±0.4 and 16.1±0.5 mm at 5.6 d (n = 19), indicating that microgravity conditions barely affected elongation growth of rice shoots. In the previous space experiment [33], elongation growth of rice coleoptiles was stimulated by approximately 5% in space compared with on the ground, in which plants were cultivated independently in space (in orbit) and on the ground. In contrast, the present space experiment cultivated rice seedlings simultaneously in orbit under both artificial 1 *g* and microgravity conditions. The discrepancy in the effect of microgravity on growth may be due to different cultivation systems including incubators and cultivation containers, and to different cultivation conditions such as temperature and gas environments.

In the present space experiment, we prepared 8 culture dishes (total 200 caryopses) for each day and each gravity condition. After the cultivation, plant materials were kept frozen in culture dish on the ISS and during the flight aboard Space Shuttle and the transportation to the laboratory. The number of recovered shoots was approximately 90% of planted caryopses for 4 d seedlings, approximately 70% for 5.3 d seedlings, and approximately 50% for 5.6 d seedlings. The 5.3 d and 5.6 d samples, particularly 5.6 d sample, contained a number of broken shoots. The 4 d seedlings that had short shoots did not contain broken shoots. Therefore, we assume that the frozen long shoots may suffer physical damage (shock) during the recovery and transportation. This problem needs a further consideration.

### Mechanical properties of the cell wall

The mechanical properties of the cell wall of rice shoots were measured by the stress-relaxation method [26]. The T_0_, a stress-relaxation parameter, is associated with viscosity according to the theoretical Maxwell viscoelastic model [26, 34, 35]. Therefore, T_0_ has been considered to represent the viscous nature of cell walls: an increase in T_0_ indicates that the cell walls have become more viscous or stiff, whereas a decrease indicates that they have become less viscous or looser. Indeed, changes in T_0_ have been reported to correlate with the capacity of cell walls to extend (cell wall loosening) in growing plant stems [34, 35].

The T_0_ value at 4 d of rice shoots grown under microgravity was almost comparable to that of shoots grown under artificial 1 *g* conditions (Table 1). However, T_0_ values of microgravity-grown shoots were significantly lower than those of 1 *g*-grown shoots at both 5.3 d and 5.6 d, although the values increased during the cultivation period (Table 1). In addition, the analysis of the significance of differences among each gravity condition by the Tukey’s test showed that T_0_ values of 1 *g*- and microgravity-grown shoots significantly increased from 4 d to 5.3 d (P < 0.05), but no significant differences were observed between 5.3 d and 5.6 d. The Tukey’s test also showed that at both 5.3 d and 5.6 d, T_0_ values of microgravity-grown shoots were significantly lower than those of 1 *g*-grown shoots (P < 0.05) similar to the result of the Student's *t*-test. These results suggest that shoot cell walls became stiff over the course of the growth period and that this cell wall stiffening was suppressed in the microgravity environment. The present results are consistent with the results of the previous space experiment, in which cell wall extensibility remained higher in space-grown rice coleoptiles than in ground-grown ones [33].

**Table 1:** Mechanical property of cell walls of rice shoots.

	Minimum stress-relaxation time (ms)		
Conditions	4 d	5.3 d	5.6 d
1 *g*	35.9±1.5	46.4±0.7	49.1±1.5
Microgravity	34.4±1.2	39.7±1.2*	41.9±1.2*

Data are means ± SE (n = 19, 30, and 17 for 4 d, 5.3 d, and 5.6 d, respectively).

*Mean values significantly different between on-orbit (artificial) 1 *g* and microgravity conditions (Student's *t*-test, P < 0.01).

In the present space experiment, we initially planned to harvest plant materials at 1–d intervals from days 4 to 6, but operational limitations in orbit constrained sampling to days 4, 5.3, and 5.6. Because the duration between 5.3 d and 5.6 d was short, and because microgravity showed similar effects on the mechanical property of cell walls at 5.3 d and 5.6 d, we used 5.3 d shoots for the analyses of cell wall constituents and 5.6 d shoots for the analyses of enzyme activities and of gene expression levels.

### Amounts of cell wall constituents

The cell walls of growing plant tissues consist of cell wall polysaccharides, proteins and phenolic substances [5]. The cell wall polysaccharides of rice shoots were fractionated into matrix polysaccharides and cellulose (Table 2) and found to consist of 54–60% matrix polysaccharides and 40–46% cellulose. The amount of matrix polysaccharides and cellulose increased around 3 times from 4 d to 5.3 d, and microgravity did not influence these amounts on either 4 d or 5.3 d (Table 2). Indeed, microgravity was previously found to barely affect the net amount (amount per organ) of cell wall polysaccharides in rice coleoptiles [33]. Similarly, hypergravity conditions (gravitational forces of more than 1 *g*) also had little effect on the net amount of cell wall polysaccharides in stem organs, although the amount of polysaccharides per unit of stem length was increased [23, 36].

**Table 2:** Amounts of cell wall polysaccharides in rice shoots.

		Sugar content (μg/shoot)	
Day	Conditions	MP	Cellulose
4 d	1 *g*	39.2±1.1	27.2±1.5
	Microgravity	39.5±0.5	27.9±0.8
5.3 d	1 *g*	104.3±3.9	89.8±2.6
	Microgravity	112.6±2.0	97.1±1.6

The sugar content in each fraction was determined by the phenol-sulfuric acid method. Data are means ± SE from three independent samples. MP, matrix polysaccharides. Statistically significant difference between on-orbit (artificial) 1 *g* and microgravity conditions was not detected.

**Fig 2:**
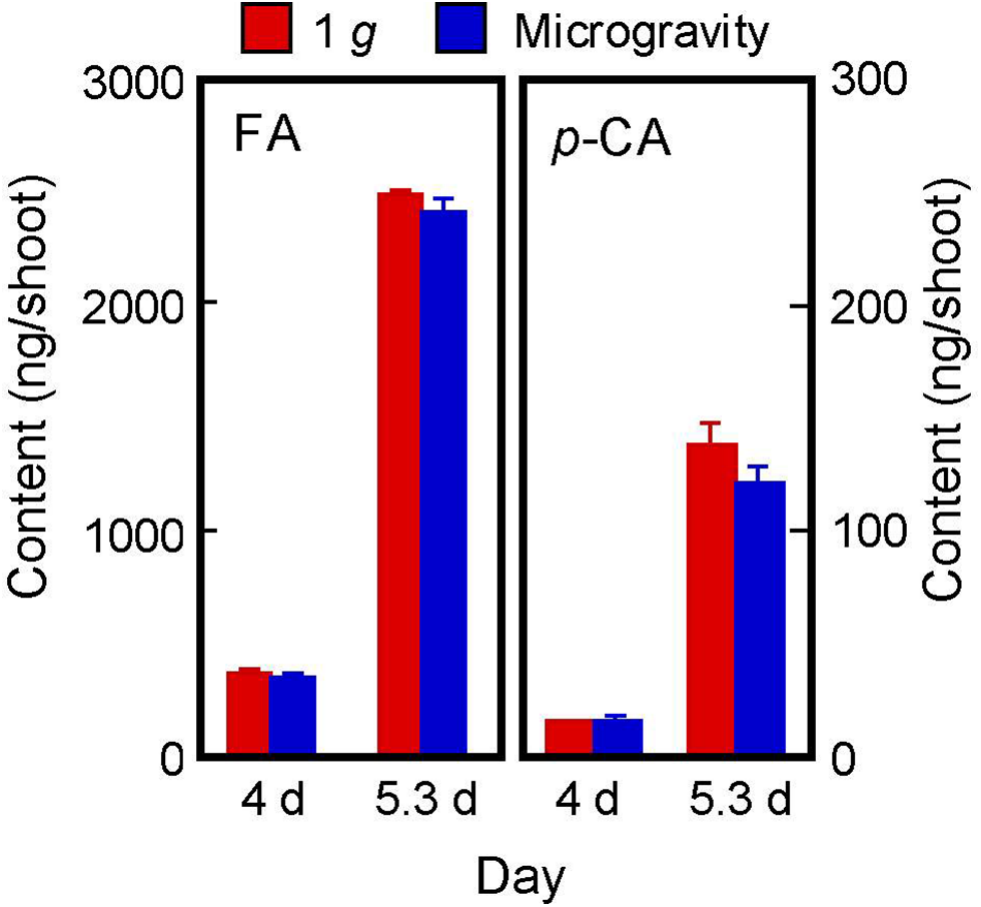
Amounts of FA and *p*-coumaric acid (*p*-CA) in the cell walls of rice shoots. FA and *p*-coumaric acid were analyzed by HPLC on a reversed-phase column. The figure shows means ± SE from three independent samples. As to FA and *p*-coumaric acid contents, statistically significant difference between artificial (on-orbit) 1 *g* and microgravity conditions was not detected.

In Type II cell walls (found in gramineous plants), phenolic acid monomers such as FA and *p*-coumaric acid are linked by esters to arabinoxylans, and some FA residues undergo a coupling reaction to produce DFA [5, 10]. Although the amount of *p*-coumaric acid was about 1/20 of that of FA, both compounds increased dramatically from 4 d to 5.3 d (Fig 2). The amounts of FA and *p*-coumaric acid at 4 d and 5.3 d were almost the same in shoots grown in 1 *g* and microgravity conditions (Fig 2), suggesting that a microgravity environment did not affect the formation of cell wall-bound phenolic acid monomers.

**Fig 3:**
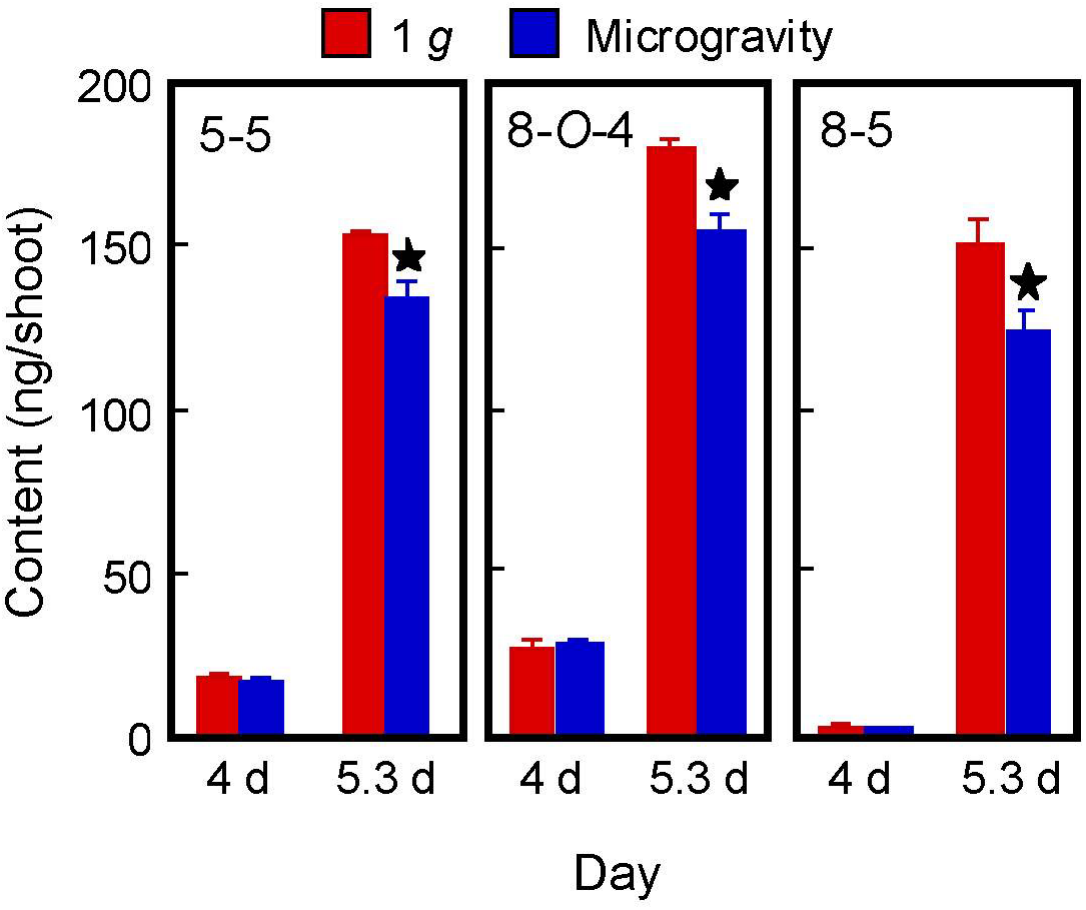
Amounts of DFA isomers in the cell walls of rice shoots. DFA isomers were analyzed by HPLC on a reversed-phase column. The figure shows means ± SE from three independent samples. *Mean values significantly different between artificial (on-orbit) 1 *g* and microgravity conditions (Student's *t*-test, P < 0.05).

Our previous study showed that the cell walls of etiolated (dark-grown) rice shoots contained three major DFA isomers: 5–5, 8-*O*-4, and 8–5 (8–5 benzofuran) forms [20]. A similar distribution of DFA isomers has been observed in the cell walls of wheat shoots [37, 38]. The amounts per shoot of these three DFA isomers increased substantially from 4 d to 5.3 d (Fig 3), as did FA content (Fig 2). The amount of DFA isomers in microgravity-grown shoots was significantly lower than in 1 *g*-grown shoots on 5.3 d, while the amounts were almost the same in shoots from both conditions on 4 d (Fig 3). This indicates that a microgravity environment reduced DFA formation, that is the coupling of FA residues, in shoot cell walls. While gravitational conditions did not affect the amount of cell wall polysaccharides (Table 2), the concentration of DFA in cell walls was lower in microgravity-grown shoots than in 1 *g*-grown shoots. In cell walls of gramineous shoots, the decrease in the amount of DFA contributes in maintaining the cell wall extensible or loosened [12, 14, 15, 39]. Therefore, decreased ferulate network formation probably contributes to keeping the cell wall loose under microgravity conditions. In contrast, hypergravity treatment increased DFA content in cell walls of wheat shoots [23].

In addition to ester-linked phenolic acids, the cell walls of terrestrial plants contain lignin. Lignin is a complex phenolic polymer with a high molecular mass and formed by the polymerization of hydroxycinnamate monomers. Lignin thus forms a complex net of cross-links among cell wall components and secures them. Hypergravity has been shown to increase the lignin content of stem organs [24, 40, 41]. We therefore analyzed lignin content in our space-grown rice shoots. As shown in Table 3, the lignin content increased dramatically from 4 d to 5.3 d. Similar to DFA isomers, the amount of lignin in microgravity-grown shoots was significantly lower than in 1 *g*-grown shoots on 5.3 d, although the amounts were almost the same in shoots from both conditions on 4 d (Table 3). These results suggest that, as with DFA, a microgravity environment reduced lignin formation and that the reduced amount of lignin may contribute to suppressing cell wall stiffening under microgravity. In contrast to our results, lignin content was almost the same in wheat seedlings grown on the ground and in microgravity conditions in space, when seedlings were cultivated under light irradiation [42]. This result suggests that light irradiation may negate the effects of microgravity on the lignin formation.

**Table 3:** Amounts of lignin in cell walls of rice shoots.

	Lignin content (μg/shoot)	
Day	1 *g*	Microgravity
4 d	0.60±0.08	0.61±0.06
5.3 d	5.37±0.15	4.67±0.07*

Data are means ± SE from three independent samples.

*Mean values significantly different between on-orbit (artificial) 1 *g* and microgravity conditions (Student's *t*-test, P < 0.05).

### Activity of PAL and CW-PRX

The activity of PAL has been shown to be involved in regulating levels of wall-bound phenolic acid monomers in some gramineous shoots [13–15]. Moreover, the treatment with an inhibitor of PAL decreased the amount of wall-bound FA in maize cell suspensions [8]. In the present study, PAL activity in rice shoots increased progressively from 4 d to 5.6 d (Table 4), as observed in a previous study [20]. The PAL activities in microgravity-grown shoots were almost comparable to those in artificial 1 *g*-grown shoots at both 4 d and 5.6 d (Table 4). These results are consistent with the changes in the amounts of phenolic acid monomers such as FA and *p*-coumaric acid in shoots in both gravitational conditions (Fig 2).

**Table 4:** Activity of PAL in rice shoots.

	Activity (ng CA/shoot)	
Day	1 *g*	Microgravity
4 d	64±27	48±15
5.6 d	845±109	834±104

Activity is expressed as the amounts of *t*-cinnamic acid (CA) produced during the assay. Data are means ± SE from three independent samples. Statistically significant difference between on-orbit (artificial) 1 *g* and microgravity conditions was not detected.

**Fig 4:**
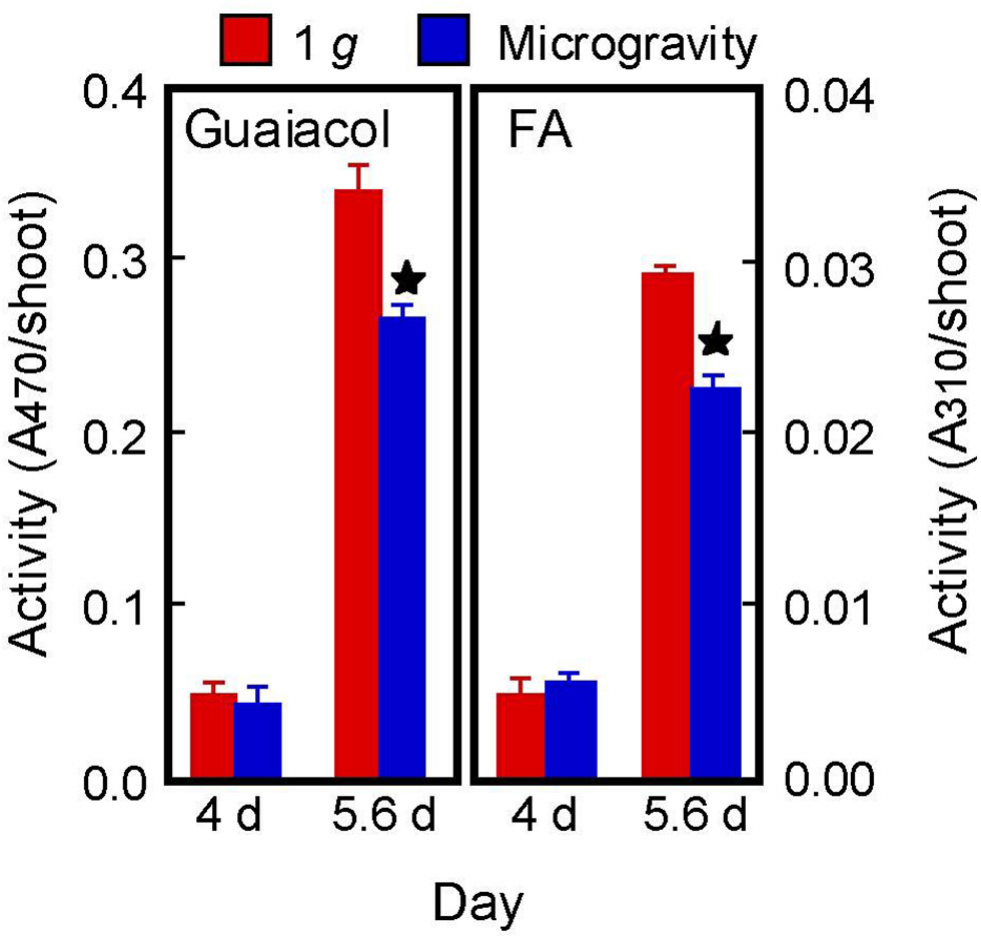
Activity of CW-PRX in rice shoots measured using two substrates, guaiacol and FA. Activity is expressed as the increase in the absorbance at 470 nm for guaiacol and as the decrease in the absorbance at 310 nm for FA. The figure shows means ± SE from three independent samples. *Mean values significantly different between artificial (on-orbit) 1 *g* and microgravity conditions (Student's *t*-test, P < 0.05).

The CW-PRX has been thought to catalyze the coupling reaction of FA to produce DFA [10, 18]. CW-PRX might also be involved in lignin formation, particularly polymerization of lignin [18, 43]. In our previous study, hypergravity treatment increased lignin content and CW-PRX activity in stems of azuki bean seedlings [24]. CW-PRX activity may thus be a crucial factor in the regulation of DFA and lignin formation under altered gravity conditions. In the present study, CW-PRX activity was measured using two substrates, guaiacol and FA. As with PAL (Table 4), the activity of CW-PRX increased substantially from 4 d to 5.6 d, irrespective of substrates, but the increases in CW-PRX activity in microgravity-grown shoots were significantly smaller than those in 1 *g*-grown shoots (Fig 4). The amounts of DFA isomers (Fig 3) and lignin (Table 3) changed in parallel with CW-PRX activity. These results suggest that suppression of the increase in CW-PRX activity may be involved in the reduction of DFA and lignin formation under microgravity conditions. We have reported that both CW-PRX activity and lignin content increased gradually from the upper, immature (growing) region toward the basal, mature region of azuki bean stems and that there was a close correlation between the lignin content and the enzyme activity [24]. Furthermore, the increase in CW-PRX activity was associated with the cessation of growth of rice coleoptiles [20]. Since CW-PRX is responsible for making firm cell wall architecture by promoting the formation of intra-network structures such as ferulate networks and lignin, an important role of CW-PRX may be to lead the cell wall development such as the transition from a flexible, immature wall to the rigid, mature wall.

### Expression of CW-PRX genes

**Fig 5:**
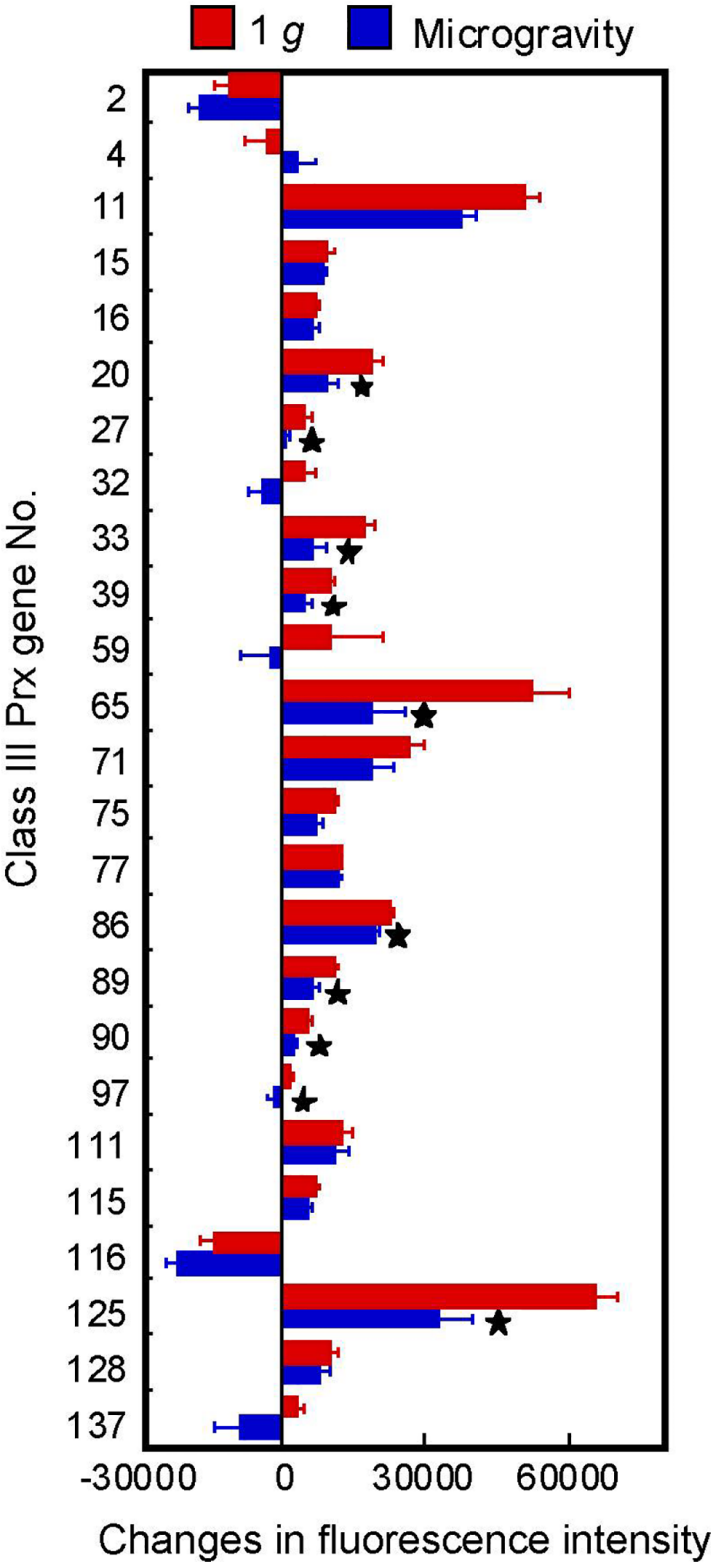
Changes in the expression levels of class III peroxidase genes in rice shoots. The difference in fluorescence intensity between 4 d and 5.6 d was calculated using the data from S1 Table for peroxidase genes that had fluorescence intensities above 10,000. The figure shows means ± SE from four independent samples. *Mean values significantly different between artificial (on-orbit) 1 *g* and microgravity conditions (Student's *t*-test, P < 0.05).

Peroxidases in plant cells are divided into two major groups, class I and class III. Class I and III are considered as intracellular peroxidases and secretory peroxidases, respectively [18, 31]. CW-PRX belongs to the class III peroxidases. Class III peroxidases usually form large multigenic families and the rice genome contains 138 class III peroxidase genes [31]. To investigate the expression levels of CW-PRX, we analyzed class III peroxidase gene expression using microarray analysis data. Of 138 candidate genes, signals of 117 genes were detected, but the fluorescence intensity values of many genes were negligible. Twenty-five genes had a high value of fluorescence intensity (more than 10,000) and the intensity values of these genes changed during the growth period (S1 Table). Therefore, we calculated the difference (increase or decrease) in fluorescence intensity between 4 d and 5.6 d (Fig 5). The intensity values of most of the genes increased from 4 d to 5.6 d. In particularly, the values of genes no. 11, no. 65 and no. 125 increased greatly during this period and their values were lowered under microgravity conditions. In the previous study, we analyzed the expression levels of rice class III peroxidase genes during shoot development by the quantitative real-time RT-PCR [20]. We have designed a set of primers based on the conserved region of six class III peroxidase genes. The expression levels measured using such primers showed a good accordance with the CW-PRX activity in rice shoots [20]. Using these primers, the expression level of peroxidase genes on 5.6 d was analyzed. The expression level in shoots grown under microgravity conditions (72±7.0%, n = 4) was significantly lower than that grown under artificial 1 *g* conditions (100±5.1%, n = 4). These results indicate that a microgravity environment lowered the expression levels of class III peroxidase genes in rice shoots. In a recent report, it was shown that a microgravity environment reduced the expression levels of some class III peroxidase genes in *Arabidopsis* seedlings and the reduction of peroxidase gene expression was involved in reduced root hair growth under microgravity conditions [44]. From these findings, we assume that a microgravity environment represses the expression of certain class III peroxidase genes in shoots of terrestrial plants and that the modification of gene expression levels may be involved in the modulation of CW-PRX activity under microgravity conditions.

In contrast to microgravity, hypergravity treatment has been shown to increase expression levels of a class III peroxidase gene and genes related to the lignin biosynthesis in *Arabidopsis* inflorescence stems [45]. Therefore, we also analyzed the expression levels of lignin-related genes using data of the microarray analysis (S2 Table). However, a microgravity environment for several days had minimal effect on the expression levels of lignin-related genes in rice shoots (S2 Table).

Plants are constantly exposed to environmental stimuli such as gravity, light and water status, and they continually respond to them. Various compounds in plant cells act as signaling molecules that mediate responses to stimuli. Tamaoki et al. [45] reported that hypergravity treatment stimulated endogenous auxin accumulation in *Arabidopsis* inflorescence stems, suggesting that auxin dynamics may be involved in plant responses against altered gravitational force. In addition, it has been shown that hydrogen peroxide, a requisite substance for peroxidase reaction, functions as a signaling molecule in plants and it is involved in gravitropism [46]. Indeed, exogenously applied hydrogen peroxide inhibited gravitropism and induced horizontal curvature of primary roots of grass pea seedlings [47]. Therefore, the hydrogen peroxide signaling may also be involved in responses against altered gravity conditions such as microgravity and hypergravity. In a recent report, it was shown that the level of hydrogen peroxide in callus cell cultures of *Arabidopsis* changed in transient microgravity phases during parabolic flights [48]. These findings suggest that long term microgravity environment might alter the cellular hydrogen peroxide level by which the downstream signaling events including gene expression would be modulated.

## Conclusions

The results of the present study showed, for the first time, that a microgravity environment could suppress the formation of ferulate networks in cell walls of rice shoots by modifying the expression levels of certain class III peroxidase genes and thus the activity of CW-PRX. The lowered activity of CW-PRX was also associated with a decrease in lignin formation. Reduced formation of structures within the cell wall, via the effects on the ferulate network and lignin, may be involved in keeping the cell wall loose under microgravity conditions. Opposite changes have been observed in plant shoots grown under hypergravity conditions [22–24]. This suggests that the network structures mediated by hydroxycinnamates may serve a load-bearing function and contribute to the rigidity of the cell wall against the force of gravity. Taken together, the present results provide evidence that gravitational stimuli modify the metabolism of cell wall hydroxycinnamates and thus regulate the mechanical strength of the cell wall of gramineous plants.

## Supporting Information

S1 Fig Experimental devices used in Ferulate experiment. Upper panels show the culture dish and rice seeds (caryopses) planted in the dish. Lower panels show a Measurement Experiment Unit (MEU) and the Cell Biology Experiment Facility (CBEF). Culture dishes were installed into MEUs and then stored at 2°C. On the ISS, cold-stored MEUs were transferred to the CBEF. The CBEF is an incubator with two compartments: a microgravity part and an artificial gravity part, in which a centrifuge produces 1 *g* conditions.
(TIF)

S2 Fig Changes in the temperature of culture dishes after transfer to the CBEF. Culture dishes placed in MEUs were first stored at 2°C and then installed into the microgravity and artificial gravity compartments of the CBEF in the Kibo Module of the ISS. Temperature was recorded in a button battery-type temperature logger attached to the culture dish.
(TIF)

S1 Table Expression levels of class III peroxidase genes in rice shoots. Peroxidase genes with a fluorescence intensity of more than 10,000 are shown. Data are means ± SE from four independent samples. *, **Mean values significantly different between on-orbit 1 *g* and microgravity conditions on day 4 and day 5.6, respectively (Student's *t*-test, P < 0.05).
(XLS)

S2 Table Expression levels of lignin-related genes in rice shoots. Genes involved in lignin formation in rice plants were selected from the Oryzabase (http://www.shigen.nig.ac.jp/rice/oryzabase/). The expression levels of genes were analyzed using the values of fluorescence intensities obtained through a microarray analysis. Data are means ± SE from four independent samples. *, **Mean values significantly different between on-orbit 1 *g* and microgravity conditions on day 4 and day 5.6, respectively (Student's *t*-test, P < 0.05). C4H, Cinnamate 4-hydroxylase; C3H, Coumarate 3-hydroxylase; COMT. Caffeate *O*-methyltransferase; CCoAOMT, Caffeoyl CoA *O*-methyltransferase; F5H, Ferulate 5-hydroxylase; 4CL, 4-Coumarate:CoA ligase; CCR, Cinnamoyl-CoA reductase; CAD, Cinnamyl alcohol dehydrogenase.
(XLS)
