# Supplementary figures and images for: Suppression of Hydroxycinnamate Network Formation in Cell Walls of Rice Shoots Grown under Microgravity Conditions in Space
Source: PLoS One. 2015 Sep 17;10(9):e0137992. doi: 10.1371/journal.pone.0137992 (PMC4574559; doi:10.1371/journal.pone.0137992)

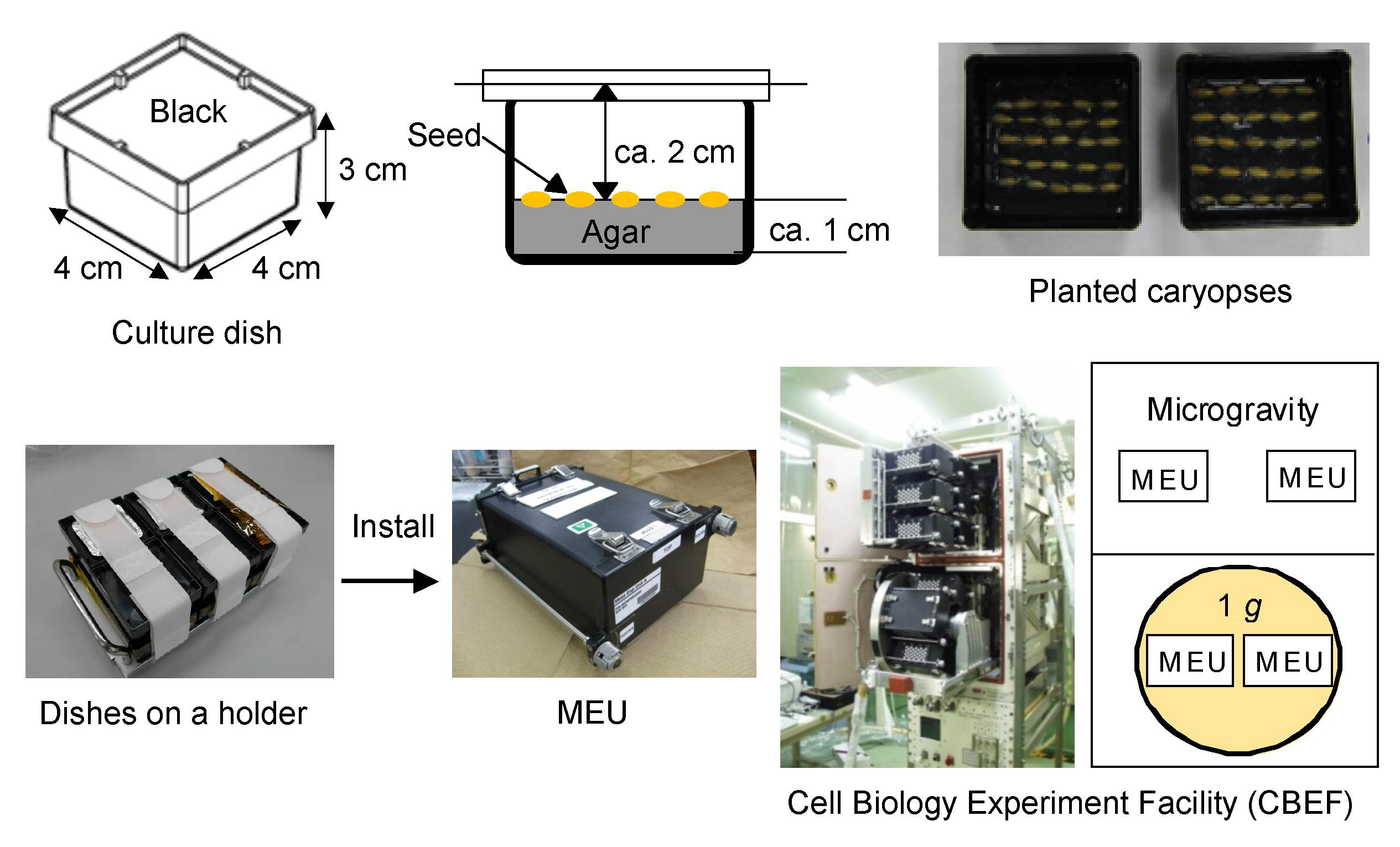

Supplement: S1 Fig — Upper panels show the culture dish and rice seeds (caryopses) planted in the dish. Lower panels show a Measurement Experiment Unit (MEU) and the Cell Biology Experiment Facility (CBEF). Culture dishes were installed into MEUs and then stored at 2°C. On the ISS, cold-stored MEUs were transferred to the CBEF. The CBEF is an incubator with two compartments: a microgravity part and an artificial gravity part, in which a centrifuge produces 1 g conditions. (TIF) [file pone.0137992.s001.tif]

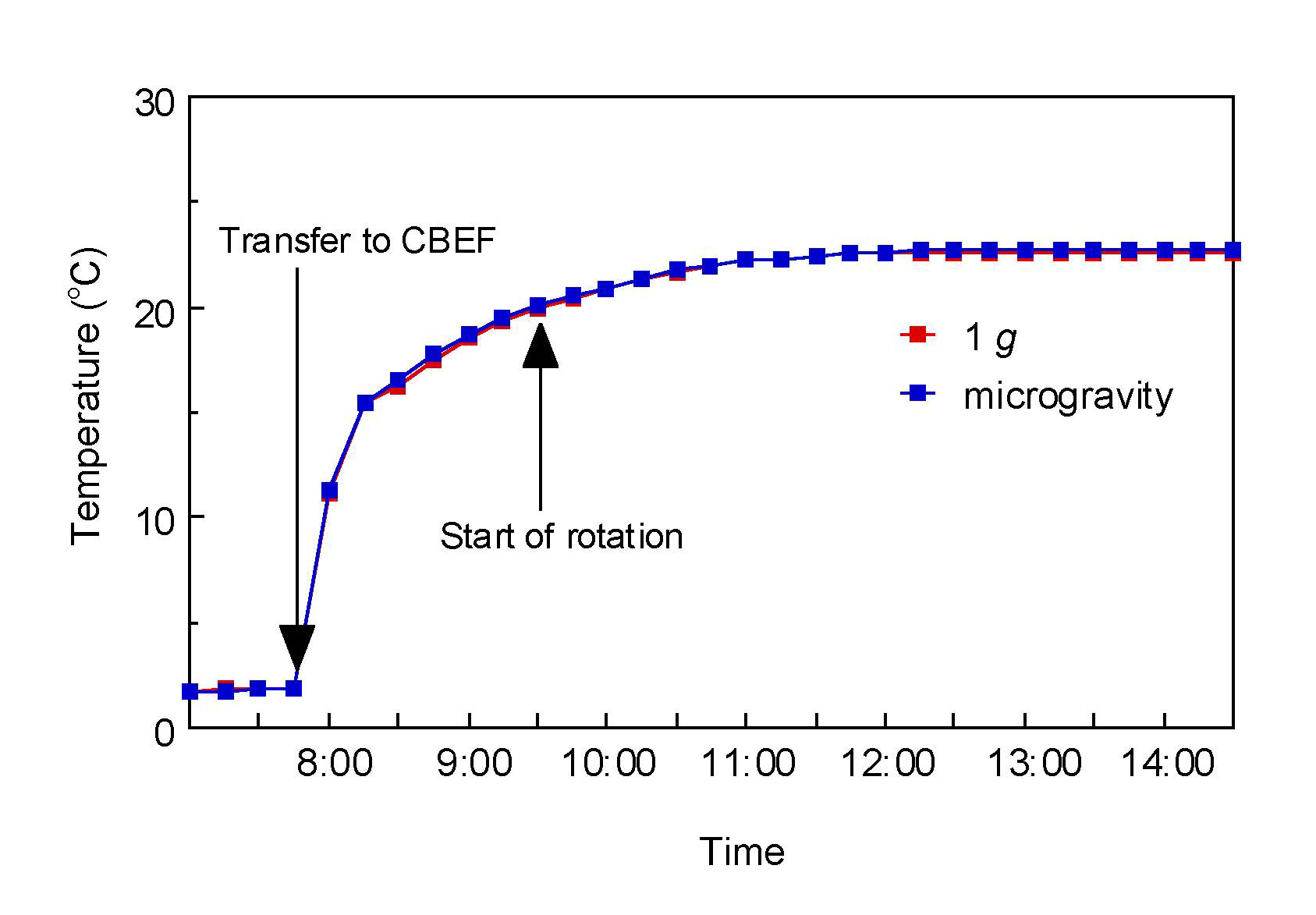

Supplement: S2 Fig — Culture dishes placed in MEUs were first stored at 2°C and then installed into the microgravity and artificial gravity compartments of the CBEF in the Kibo Module of the ISS. Temperature was recorded in a button battery-type temperature logger attached to the culture dish. (TIF) [file pone.0137992.s002.tif]
